# Exploring immunological alterations of B cells in peripheral immunity via single-cell RNA sequencing: insights into primary membranous nephropathy

**DOI:** 10.3389/fimmu.2025.1622395

**Published:** 2025-08-19

**Authors:** Fang Lu, Si Chen, Honglei Guo, Qing Li, Lin Wu, Ying Pan, Yangfan Wu, Hua Shu, Simeng Liu, Bo Zhang, Huijuan Mao, Changying Xing, Hongwei Liang, Suyan Duan, Yanggang Yuan

**Affiliations:** ^1^ Department of Nephrology, The First Affiliated Hospital with Nanjing Medical University, Nanjing Medical University, Nanjing, China; ^2^ School of Life Science and Technology, China Pharmaceutical University, Nanjing, Jiangsu, China

**Keywords:** ScRNA-seq, PBMC, PMN, galectin-9, B cell

## Abstract

**Background:**

Primary Membranous Nephropathy (PMN) is characterized by dysregulated immune responses, with B cells playing critical roles in disease pathogenesis. However, the immunopathogenic mechanisms underlying B cell involvement in PMN remain elusive.

**Methods:**

We employed single-cell RNA sequencing on peripheral blood mononuclear cell samples (PBMC) obtained from 6 patients with PMN and 3 healthy controls (NC) to explore the transformation of B cells and their interaction with immune cells.

**Results:**

Compared with NC, the most significant alterations were in plasma cells and regulatory B (Breg) cells in PMN patients. Within plasma cells, Subcluster 0 was increased in PMN patients and exhibited enhanced autoimmunity. Breg subset B10 cells were elevated in PMN patients and displayed increased immune regulatory capacity, marked by enhanced cytokine and interleukin-10 production. B cell activating factor (BAFF) and galectin-9, which were secreted by CD14 monocyte, as potential regulators of plasma and Breg cells activity. Additionally, serum galectin-9 levels increased in PMN patients and showed a correlation with proteinuria and renal function in PMN.

**Conclusions:**

We reveal novel insights into the heterogeneity and functional diversity of B cells in patients with PMN. And revealed distinct roles for subgroup 0 plasma cells and B10 Breg cells in the pathogenesis of PMN. Furthermore, targeting B cells, such as galectin-9, presents promising opportunities for modulating the immune response in patients with PMN.

## Introduction

Membranous nephropathy (MN), as a leading cause of nephrotic syndrome in adults, is characterized by extensive alterations in podocyte structure and function, glomerular basement membrane (GBM) expansion and diffuse subepithelial immune deposition (1). The pathogenesis network of MN involves a variety of antigen-antibody systems, genes and cytokines associated with immune response, progression and recovery (2). Approximately 80% of cases, known as primary membranous nephropathy (PMN), is regarded as an organ-specific autoimmune disease in which the identification of a growing range of podocyte autoantigens, such as phospholipase A2 receptor (PLA2R), neutral endopeptidase (NEP), and thrombospondin type-1 domain-containing protein 7A (THSD7A). The corresponding circulating autoantibodies, especially serum anti-PLA2R antibodies (aPLA2Rab), facilitate better management in disease diagnosis, monitoring, and therapeutic interventions in PMN (3). Advancements in identifying podocyte autoantigens have significantly deepened our understanding of this disorder (2, 4). Furthermore, immune cells are key drivers of autoimmune and related organ damage, which are also profoundly immersed in the occurrence and development of PMN (5). Numerous studies have demonstrated B cell abnormalities in both peripheral blood and renal tissues of PMN patients across different stages of the immune response, encompassing self-antigen recognition and autoantibody production (4). Moreover, T cells are a key component of the effector and regulatory immune responses, supporting B cell-related responses and orchestrating inflammation and cytotoxicity, which induce kidney tissue damage in PMN (2, 6). Characterizing the dynamics of B cell and T cell populations at disease onset, remission, and relapse, and understanding their interactions to modulate disease progression remains a gap in the current knowledge of PMN (7). Consequently, a comprehensive elucidation of the distinct roles played by each immune cell subset and their intricate interplay is essential to deciphering the underlying immunopathogenic mechanisms driving PMN. The rapid development of single-cell RNA sequencing (scRNA-seq) has revolutionized our ability to study gene expression at the cellular level. This powerful technology enables researchers to identify novel cell types, refine existing classifications, and gain unprecedented insights into the intricacies of cell differentiation (8). ScRNA-seq has been employed to map the cellular landscape of kidney biopsies and urine samples from PMN patients, providing unprecedented insights into the disease’s cellular heterogeneity and molecular pathophysiology (9, 10). More recently, Gu et al. profiled the transcriptomic landscapes of blood, kidney and urine in patients with PMN using scRNA-seq, and depicted the alterations including cell compositions and cell-cell communication in PMN (11). However, the current understanding of the distribution of peripheral blood immune cells in patients with PMN is based on limited sample sizes, and the cell type-specific functional status of immune cells, particularly different subtypes of B cell, remains unclear. In addition, none of the studies has reported exploring the role of critical B cell subsets and their interaction with other immune cells in the peripheral blood of patients with PMN.

In this study, we investigated peripheral blood mononuclear cells (PBMC) from 6 biopsy-proven PMN patients and 3 healthy controls (NC) using scRNA-seq on the BD Rhapsody system. Our analysis revealed the proportions of immune cell subsets, characterized gene transcription states, and identified hub genes and pathways associated with PMN. Our high-resolution transcriptome map of immune cells during PMN occurrence and progression offers valuable insights into the immunological landscape and pathogenic immune response, potentially guiding novel therapeutic strategies.

## Materials and methods

### Ethical approval and consent

This study followed the Declaration of Helsinki and was approved by the Ethics Committee of the First Affiliated Hospital of Nanjing Medical University (Approval No.2022-SR-444). Patients and healthy adult volunteers provided consent for all experiments. All the experimental methods were carried out in accordance with the approved guidelines. Written informed consent was obtained from the patients/participants for the publication of any potentially identifiable images or data included in this article.

### Patient involvement

For scRNA-seq analysis, 6 biopsy-proven PMN patients and 3 healthy adult volunteers were enrolled at the Department of Nephropathy of the First Affiliated Hospital of Nanjing Medical University from January 2023 to January 2024. And for subsequent serum galectin-9 detection analysis, a total of 69 patients diagnosed with PMN, 10 patients with focal segmental glomerulosclerosis (FSGS), 10 patients with IgA nephropathy (IgAN), 10 patients with diabetic kidney disease (DKD), and 10 healthy adult volunteers as normal controls (NC) were enrolled. All enrolled patients were treatment-naive and had not received immunosuppressive therapy prior to blood sample collection. The inclusion criteria of PMN were as follows: (1) age≥18 years; (2) PMN is diagnosed and confirmed by renal biopsy, with concurrent nephrotic syndrome; and (3) with positive serum aPLA2Rab. Patients with secondary MN, such as those with autoimmune diseases (e.g. systemic lupus nephritis(SLE) and Sjogren syndrome), infection-related MN resulting from hepatitis B and C virus infection, or MN related to malignancies, medications, or heavy metal poisoning, were excluded. The diagnosis of kidney diseases complied with the Kidney Disease: Improving Global Outcomes (KDIGO) guidelines. All participants have signed the informed consent form.

### Clinical and pathological parameters

Baseline demographics, laboratory parameters, pathological data, and treatment at biopsy were collected in detail from the electronic medical records of enrolled patients. The eGFR for all patients was calculated using the chronic kidney disease epidemiology collaboration (CKD-EPIcr) formula. Mean arterial pressure (MAP) was calculated as diastolic blood pressure +1/3 of the pulse pressure.

### Blood sample preparation

**Figure 1:**
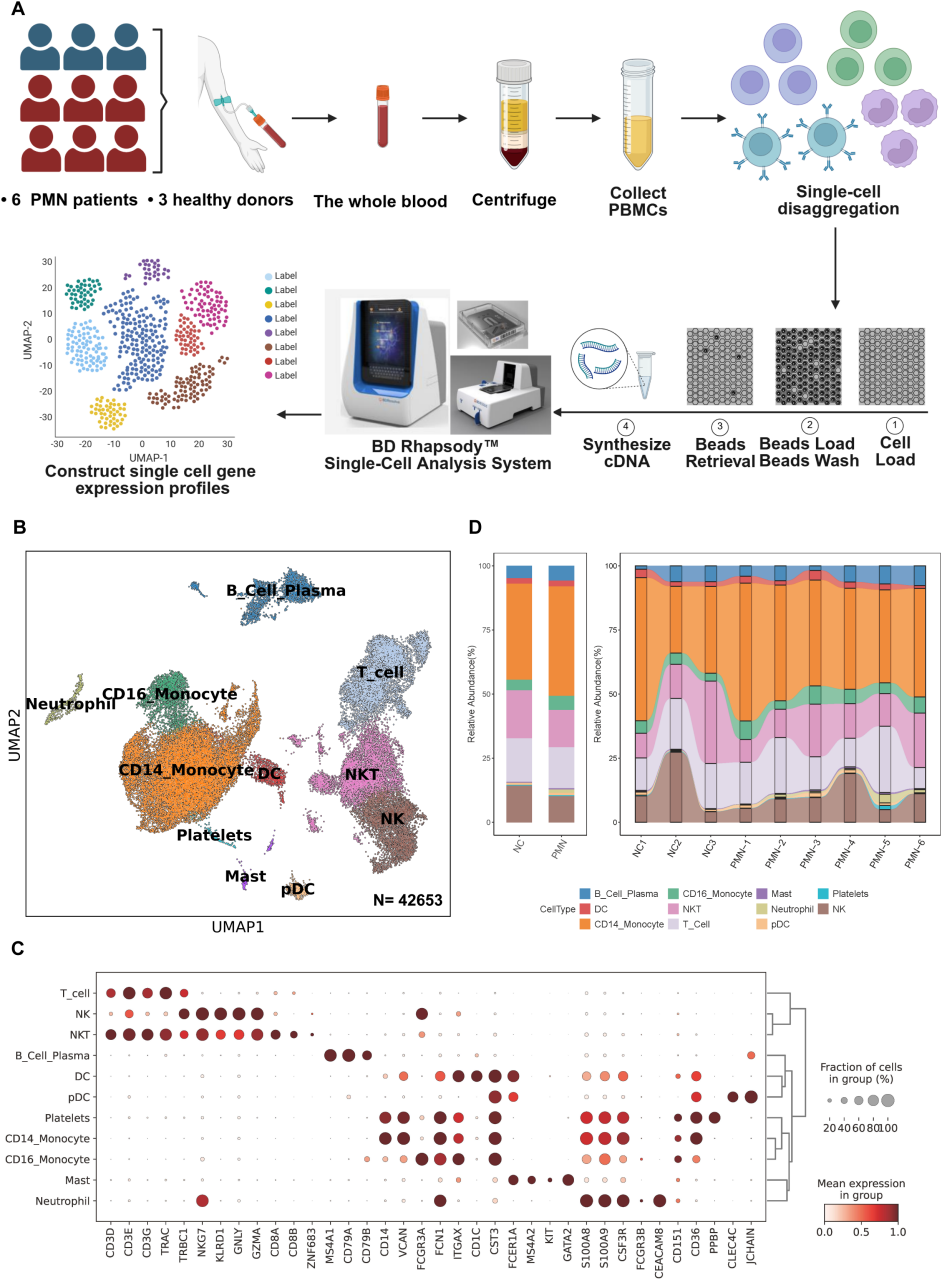
scRNA-seq analysis of PBMC from patients with PMN and normal controls. **(A)** Schematic of the experimental workflow. Blood samples were collected from patients with PMN (n=6) or healthy control (NC) subjects (n=3). PBMC were obtained and further performed single-cell RNA sequencing using the BD Rhapsody system **(B)** UMAP plot for 42653 PBMC cells, annotated with 11 cell types based on differential gene expression analysis using the Seurat FindAllMarkers function. **(C)** Dotplot of selected marker genes that identified the clusters generated by UMAP plotting. **(D)** The stacked bar chart showing the abundance of different cell types between groups and individuals. PMN, primary membranous nephropathy; UMAP, uniform manifold approximation and projection; NK, nature killer cells; NKT, nature killer T cells; Mast, mast cells; DC, dendritic cells; pDC, plasmacytoid dendritic cell. Cell types were defined.

The study was performed as shown in **Figure 1A**. Peripheral blood samples were collected after an overnight fast, prior to any medication intake. The blood was obtained and collected in heparin anticoagulant tubes. Fresh blood was separated to obtain peripheral blood mononuclear cells (PBMC) by using Histopaque-10771 (Sigma Aldrich, catalog No.10771-6 ×100 mL) according to the manufacturer’s protocol.

### Single-cell RNA sequencing

Single-cell transcriptome information was captured using the BD Rhapsody system. The single-cell suspension was randomly assigned to 200,000 micropores by a limited dilution method. The beads with oligonucleotide barcodes were added to the saturated state and paired with cells in the micropores. The cells were cleaved in micropores to hybridize mRNA molecules and the oligonucleotides on the beads were captured by bar code. After reverse transcription, ExoI digestion was performed in a test tube. During cDNA synthesis, a unique molecular identifier (UMI) was bound to each cDNA molecule at the 5’ end to indicate the origin of the cell. The BD Rhapsody single-cell full transcriptome workflow includes random primer and extension (RPE), RPE amplification PCR and WTA index PCR to prepare a full transcriptome library. The high-sensitivity DNA chip (Agilent) on the bioanalyzer 2200 and the qubit high-sensitivity DNA analysis (Thermo Fisher Scientific) were used to quantify the library. The sequencing was done on a 150-bp paired-end run by an Illumina sequencer (Illumina, San Diego, CA).

### Single-cell RNA statistical analysis

Fastp and default parameters are used to filter the adapter sequence and delete low-quality reads to obtain clean data. With the application UMI-tools (12), the single cell transcriptome recognizes the whitelist of cell barcodes. Then, the data were mapped to the human genome (Ensemble version 91) by STAR mapping (13). The UMI-tools standard pipeline was used to obtain the UMI counts for each sample. Cell with more than 200 expressed genes and mitochondria UMI rate below 20% passed the cell quality filtering and mitochondria genes. Seurat (v.5.1.0) was used to perform cell normalization and regression to obtain scaled data. After principal component analysis (PCA) using a standard of the top 2000 highly variable genes, data was processed integration and batch correction by Harmony (v.1.2.0). Dimensional reduction by uniform manifold approximation and projection (UMAP) (dims = 1:30). Cell types were defined based on differential gene expression as determined by the Seurat FindAllMarkers function.

### Differential gene expression analysis

Differential gene expression analysis was performed by a pseudobulk approach using the bulk RNA-Seq tool edgeR (v.4.2.1). Pseudobulk methods outperform mixed models and limit pseudoreplication bias. For each cell subtype, read counts were summed across each sample to create a pseudobulk count matrix. edgeR was applied, using a likelihood ratio test corrected on batch effect with an additional fit of a Gamma-Poisson generalized linear model (GLM). *P* values were adjusted using Benjamini-Hochberg method, and genes with False Discovery Rate (FDR) ≤ 0.05 were selected. Additional filtering was applied with an absolute log_2_fold-change (log2FC) ≥ 0.5.

### Gene ontology pathway analysis

The ClusterProfile (v.4.12.0) R package’s EnrichGO function was used to analyze Gene Ontology (GO) terms. Pathways with adjusted *P* < 0.05 and q <0.25 were considered statistically significant.

### Cellular pathway score

The hallmark gene set and C5 (ontology gene sets) gene set in the Human Molecular Signatures Database (MSigDB) were selected as the pathway (14). The level of gene set enrichment in each cell was assessed using the irGSEA (v.3.3.0) R package, and the calculation of the enrichment scoring method was set as UCell. This package was used to score individual cells and to generate numerous gene set enrichment score matrices. irGSEA. Density scatterplot was used to visualize the results. The stat_compare_means function was used to calculate the difference between the two groups using the t.test method. Gene Set Variation Analysis (GSVA) was used to perform enrichment scores for the hallmark gene pathways (15). GSVA scores were calculated between PMN and NC groups in different cell types by using the R package limma (v.3.60.0) to identify differentially enriched pathways. Pathways with GSVA scores with |t-values| >1 were identified as significantly enriched.

### High-dimensional weighted correlation network analysis

High-Dimensional Weighted Correlation Network Analysis (hdWGCNA) was performed to identify potential genes associated with clinical and pathology index by using hdWGCNA package (v.0.3.03) (16). Genes expressed in at least 5% of cells were to construct the hdWGNCA object, and then transformed into a Metacells object. The co-expression network was constructed with a soft power of 14 for subsequent analysis. Subsequently, the ModuleEigengenes function was utilized to calculate the module feature genes (ME) on a subset of the gene expression matrix specific to each module. The GetModuleTraitCorrelation function was utilized to screen for important modules based on their correlation coefficients and P-values in relation to clinical and pathology index. The top 20 hub genes in each module were identified based on eigengene-based connectivities (kME). Hub genes were further analyzed using GlueGo in Cytoscape software to elucidate their functional relationships and pathways.

### Pseudotemporal ordering of B cells

The pseudotime trajectories of B cells were analyzed by the Monocle2 package (version 2.32.0) using DDR-Tree and default parameter. Based on pseudo-temporal analysis, the branch expression analysis model (BEAM Analysis) was used to analyze branch fate-determining genes. CytoTRACE (version 0.3.3) was also utilized to predict the order of cell differentiation states and stemness of cells (17).

### Cell communication analysis

CellChat (v1.6.1) was used to infer the cell-cell interactions across different cell types by using the expression of known ligand-receptor pairs and identifying the changes in intercellular communications (18). All cell types in PBMC were included in the analysis. The significant ligand-receptor pairs across comparisons were recognized by using the netVisual_aggregate, net_bubble, and netAnalysis_signalingRole_network functions in CellChat.

### SCENIC analysis of B cells

Single-cell regulatory network inference and clustering (SCENIC) analysis was performed using utilizing pySCENIC (v0.10.0) based on the hg38-refseq_r8 - SCENIC databases (19). Default parameters were used for the SCENIC workflow, and the raw count matrix from all the samples was used as the input. A three-step process was used for the analysis. Firstly, GRNBoost was applied to calculate the co-expression modules and evaluate the weight between transcriptional factors (TFs) and their target genes. Next, TFs with direct targets (regulons) were identified by using RcisTarget. Finally, the activity of each regulon in each cell was evaluated using AUCell. Limma was used to identify differential TFs activity between MN and NC groups with adjusted p value < 0.05 and absolute log_2_fold-change ≥ 0.5. For visualization, the average regulon activity (AUC) scores for each cell type were calculated, and a rank plot of regulons was drawn using ggplot2.

### Serum galectin-9 detection

Serum galectin-9 was quantified using an enzyme-linked immunosorbent assay (ELISA) kit (Proteintech Group Inc., CA, USA). The sandwich ELISA consisted of anti-human galectin-9 mouse monoclonal antibodies as coating antibodies and biotinylated goat anti-human galectin-9 polyclonal antibodies as detection antibodies. Both antibodies were raised using recombinant protein (1–323 residues of galectin-9). Colorimetric analysis was performed using streptavidin-conjugated horseradish peroxidase and tetramethylbenzidine. The concentration of galectin-9 was calculated using a standard curve constructed with recombinant human galectin-9 (20).

### Statistical analysis

Clinical samples in this study were collected from individuals independently. Data were presented as mean ± SD, median and interquartile range or percentage. Comparisons between groups were made using one-way analysis of variance (ANOVA), Kruskal-Walli’s test, or χ2 test as appropriate. Spearman correlations were calculated to characterize the associations between baseline characteristics and serum galectin-9 level. ROC curve analyses to evaluate the predictive accuracy of serum galectin-9 for discriminating PMN from non-PMN. *p*-value<0.05 was considered statistically significant. All statistical analyses were conducted using R statistical software.

## Results

### Single-cell transcriptomes profiling of PBMC from PMN patients

ScRNA-seq of PBMC was performed in 6 patients with PMN and 3 NC. The demographics, clinical, and pathological features of these subjects are shown in **Table 1**. PMN patients were presented with nephrotic syndrome, with a median urinary protein of 7.6 g/d (IQR 5.1, 8.2) and a serum albumin of 19.7 g/L (IQR 18.2, 23.7 g/L). Those patients’ ages ranged from 39 to 63 years and all of them were male. The estimated glomerular filtration rate (eGFR) of PMN patients ranged from 93.2 to 125.4 ml/min/1.73m^2^ (105 ± 11.8 ml/min/1.73m^2^). Besides, serum aPLA2Rab levels ranged from 81.3 to 526.1 (median 153.2) RU/ml. On light microscopy, the classical presence of PMN was detected in all the kidney specimens, with MN ranging from I to III. No crescents formation was found in the glomeruli, and the glomerular sclerosis was mild (**Table 1**). Through immunofluorescence microscopy, all PMN patients showed a strong immunoglobulin G (IgG) staining, accompanied by deposition of complement 3 and IgM. All specimens exhibited positive PLA2R staining, confirming the diagnosis of PMN.

**Table 1:** Clinical and pathological parameters of patients with PMN enrolled in scRNA-seq.

Patients	P1	P2	P3	P4	P5	P6
Clinical parameters						
Gender	Male	Male	Male	Male	Male	Male
Age (years)	41	63	57	37	39	46
Proteinuria (g/d)	8.1	7.1	3.5	8.2	10.0	5.1
Serum Alb (g/L)	19.1	17.6	18.2	28.3	20.3	23.7
Scr (μmol/L)	91.4	73.7	68.8	56.0	79.6	85.5
aPLA2Rab (RU/mL)	526.1	81.3	151.0	293.9	155.3	132.3
eGFR (ml/min/1.73 m²)	93.2	98.2	104.1	125.4	111.4	97.8
Pathological parameters						
MN stage	II-III	II	I-II	II-III	III	II
IgG deposition	2+	3+	2+	2+	2+	3+
IgA deposition	–	–	–	–	–	–
IgM deposition	1+	1+	1+	1+	1+	1+
C3 deposition	1+	1+	1+	1+	1+	1+
C1q deposition	–	–	–	–	–	–
PLA2R staining	+	+	+	+	+	+
Crescent formation	0/38	0/15	0/33	0/29	0/29	0/27
Glomerular sclerosis	3/38	2/15	1/33	3/29	1/29	5/27

P1–6 patients 1-6; eGFR estimated glomerular filtration rate; Scr serum creatine; aPLA2Rab serum anti-phospholipase A2 receptor antibody.

Fresh PBMC derived from patients with PMN and NC were used for single-cell RNA sequencing library construction with the BD Rhapsody system (**Figure 1A**). Following rigorous quality control protocols, we thoroughly collected 42653 high-quality cells, comprising 30790 cells for patients with PMN and 11863 cells for NC (**Supplementary Figure S1A**). A total of 19 major cell clusters were identified by unsupervised clustering analysis (**Supplementary Figure S1B**). Next, we defined these cell clusters by lineage-specific gene marker. 11 distinct cell types were annotated in PBMC, including T cells (CD3D), NK cells (NKG7), NKT cells (CD3D, NKG7), B cell plasma (MS4A1), DC cells (FCER1A), pDC cells (CLEC4C), platelets (CD151), CD14^+^ monocytes (CD14), CD16^+^ monocytes (FCGR3A), mast cells (KIT, GATA2), and neutrophils (FCGR3B) (**Figures 1B, C**). Furthermore, we compared the proportion of the 11 types of immune cells between the PMN group and NC (**Figure 1D**). The proportion of neutrophils was significantly higher in the PMN group than in NC (*P* = 0.024). Additionally, the proportion of CD16^+^ monocytes, mast and B cells was increased in PMN group compared with NC group; however, they didn’t reach significantly difference.

### Immune profiles of B cell subsets in PMN patients

**Figure 2:**
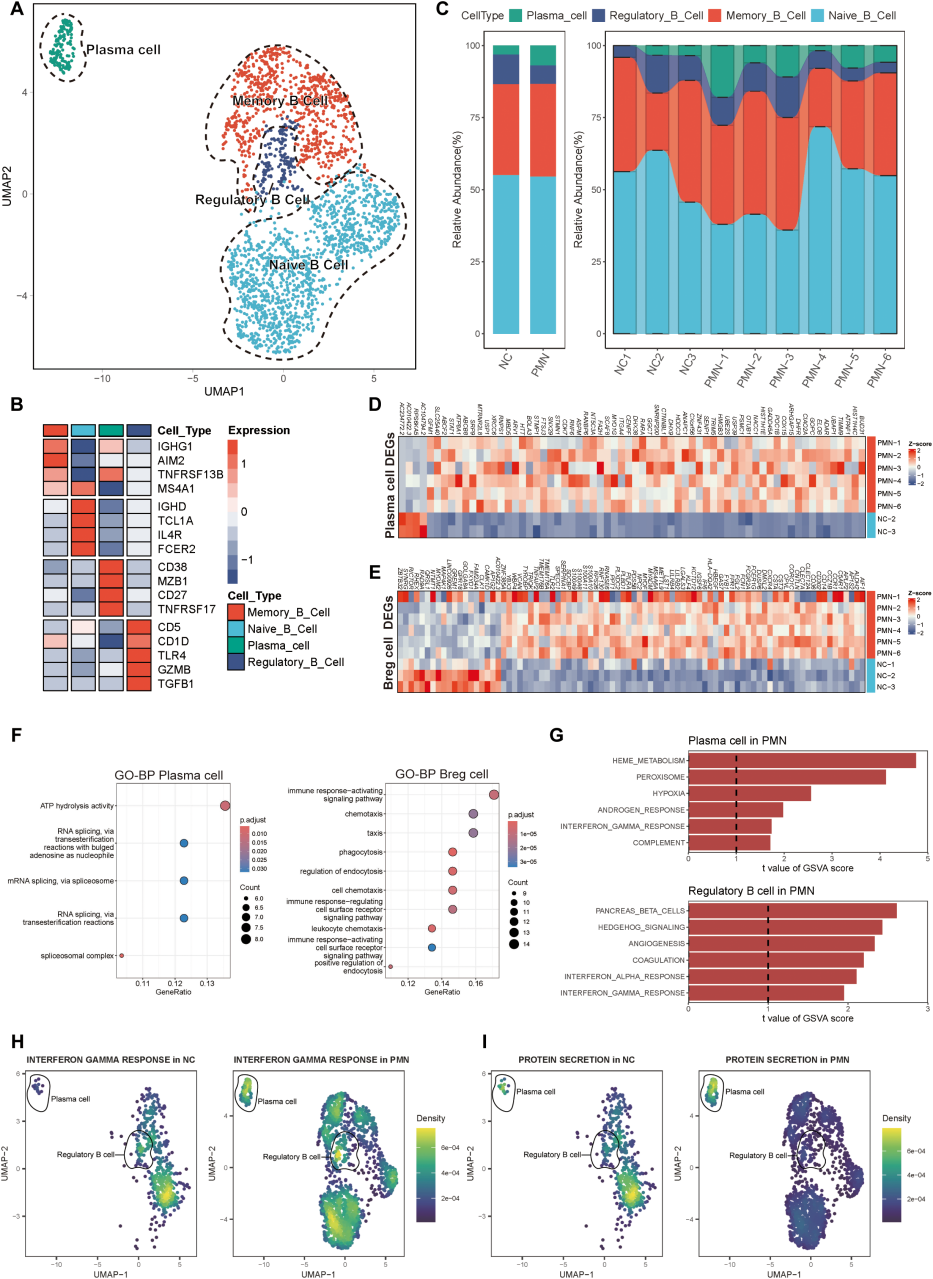
Alterations of B cells in patients with PMN. **(A)** Reclustering of the B cells and annotating according to its function. **(B)** Heatmap of selected marker genes in different subsets of B cells. Red represents high gene expression, and blue represents low gene expression. **(C)** The stacked bar chart showing the abundance of different B cells subsets between groups and individuals. **(D, E)** Heatmap of DEGs in plasma cells and Breg cells comparing the PMN patients to normal control by pseudobulk methods. The threshold for DEGs was set at an FDR of ≤ 0.05 and an absolute log2 fold-change ≥ 0.5. **(F)** GO enrichment analysis showing the biological process related pathways derived from the DEGs. Left: enrichment analysis of DEGs in plasma cells. Right: enrichment analysis of DEGs in Breg cells. Pathways with adjusted P < 0.05 and q <0.25 were considered statistically significant. **(G)** The top 6 activated pathways in PMN plasma cells and Breg cells identified via GSVA analysis. Upper: GSVA analysis in plasma cells. Lower: GSVA analysis in Breg cells. Pathways with GSVA scores with |t-values| >1 were identified as significantly enriched. **(H, I)** UMAP plot of cell pathway score in B cells between PMN and normal control groups. Gene set enrichment analysis was performed by using the irGSEA package with the UCell scoring method. **(H)** interferon γ response pathway. **(I)** Protein secretion pathway. Breg cells, regulatory B cells; DEGs, differentially expressed genes; FDR, False Discovery Rate; GO, Gene Ontology; BP, biological process; GSVA, Gene set variation analysis.

We then extracted B cells from PBMC data and labeled them according to the typical genes of B cell subsets. Four cell subtypes were identified, including the naïve B cells with canonical marker genes IGHD, TCL1A, IL4R, FCER2; memory B cells with marker genes IGHG1, AIM2, TNFRSF13B, MS4A1; regulatory B (Breg) cells with marker genes CD5, CD1D, TLR4, GZMB, TGF β1; and plasma cells marker genes CD38, MZB1, CD27, TNFRSF17 (**Figures 2A, B**). Compared with NC group, the proportion of plasma cells was significantly elevated in PMN patients (6.9% vs. 3.1%, *P* =0.048, **Figure 2C**). A total of 159 differentially expressed genes (DEGs) were identified between individuals with PMN and NC (FDR ≤ 0.05, |log2FC| ≥ 0.5, **Supplementary Figure S1C**, **Supplementary Table S1**). Breg cells (*n* = 82) and plasma cells (*n* = 64) displayed the greatest number of differentially expressed genes among all B cell subsets. The expression of RPS26 was increased in memory B cells, naïve B cells, and plasma cells, whereas the expression of AC010422.3 decreased in all subtypes of B cells of the PMN group (**Supplementary Figure S1D**). Additionally, compared with NC group, the expression of AC104794.2, RPS6KA5, AC010422.3, and AC234772.2 were significantly decreased, along with an increased expression of BUD31, HIST1H4C, ATPAF1, and TMMP9 in plasma cells of PMN patients (**Figure 2D**). Meanwhile, NTMT1, GREM1, FAM234A, and MYOM2 showed a declined expression alongside an elevated expression of LILRB4, SPECC1, WBP4, and HLA-DQA2 in Breg cells of PMN groups (**Figure 2E**). GO enrichment analyses showed DEGs mainly participated in ATP hydrolysis activity in plasma cells, and immune response-activating signaling pathway in Breg cells, indicating the different processes in the immune response of the two types of cells (**Figure 2F**). Furthermore, the GSVA revealed the activation of heme metabolism, peroxisome, hypoxia, complement, and interferon-γ response in plasma cells of PMN patients (**Figure 2G**). Hedgehog signaling, coagulation, and interferon-αresponse were also activated in Breg cells (**Figure 2G**). For naïve B cells and memory B cells, a few pathways have been observed (**Supplementary Figures S1E, F**). The cellular pathway score analysis revealed a significant increase in Interferon-γ (IFN-γ) response signaling in plasma and Breg cells and an enhanced protein secretion in plasma cells of PMN groups (compared to NC, *P <*0.01, **Figures 2H, I**, **Supplementary Figures S1G-J**).

### The role of plasma cells in the pathogenesis and progression of PMN

**Figure 3:**
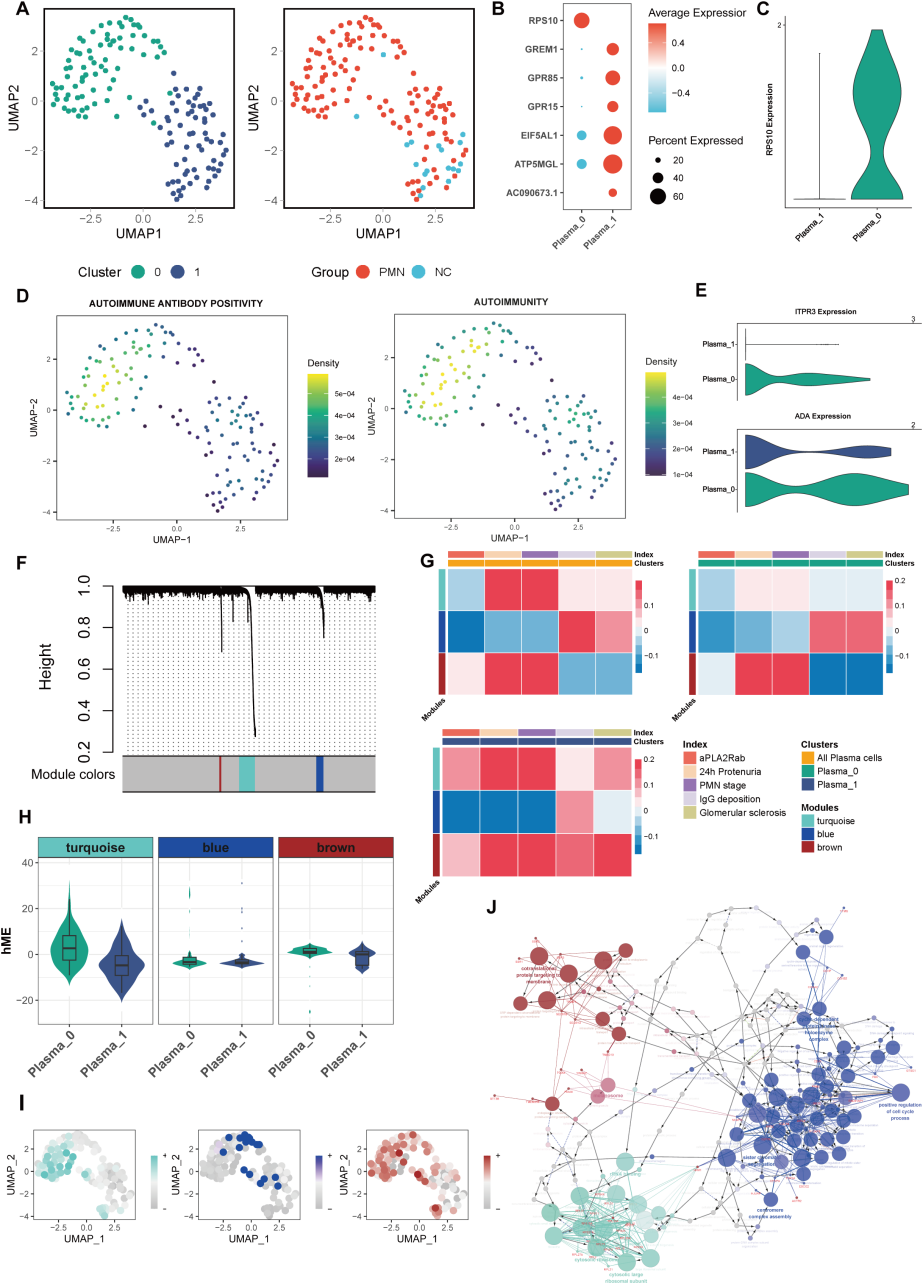
Characterization of plasma cells subclusters. **(A)** Unsupervised clustering analysis for plasma cells. **(B)** Dotplot of marker genes in each plasma subcluster. **(C)** Violin plot of plasma subcluster 0 marker gene RPS10. **(D)** UMAP plot of autoimmune antibody positivity and autoimmunity pathway scores in plasma cells. Gene set enrichment analysis was performed by using the irGSEA package with the UCell scoring method. **(E)** Violin plot of genes ITPR3 and ADA expression in 2 plasma subclusters. **(F)** hdWGCNA Dendrogram for plasma cells in PMN patients. hdWGCNA was performed to identify potential genes associated with clinical index. **(G)** Heatmap of correlation between the three modules and clinical index in plasma cells. **(H)** Violin plot of 3 modules hME expression level between 2 plasma subclusters. **(I)** UMAP plot of different module scores in plasma cells. **(J)** The network of GO terms in ClueGO of top 20 hub genes in 3 modules. Hub genes in each module were identified based on eigengene-based connectivities. The color corresponds to the respective module. hdWGCNA, High-Dimensional Weighted Correlation Network Analysis; aPLA2Rab, anti-phospholipase A2 receptor antibody; hME, harmonized module eigengenes; GO, Gene Ontology.

Plasma cells were further categorized into distinct subsets to explore their functional heterogeneity. Unsupervised clustering analysis revealed two major subpopulations: subcluster 0, characterized by high RPS10 expression, and subcluster 1, defined by elevated GREM1, GPR85, GPR15, EIF5AL1, ATP5MGL, and AC090673.1 expression (**Figures 3A-C**). The higher proportion of subcluster 1 (88.8%) was observed in NC, while a higher proportion of subcluster 0 (58.9%) was in PMN group. Compared with subcluster 1 of plasma cells, subcluster 0 cells had higher levels of positive autoimmune antibodies and autoimmunity ability, with higher expression of ITPR3 and ADA (**Figures 3D, E**). In addition, the cellular pathway score of neutrophil hemostasis, neutrophil clearance regulation of apoptotic cell clearance and regulation of glomerular filtration were significantly increased in subcluster 0 of plasma cells (**Supplementary Figures S2A–D**). In an endeavor to identify pivotal hub genes of plasma cells associated with disease activity in PMN, a comprehensive analysis using hdWGCNA of plasma cells was conducted. A total of 3 modules were identified (**Figure 3F**) after setting the soft threshold to 14 and applying the dynamic tree-cutting algorithm to merge similar gene modules. In subcluster 1 of plasma cells, genes within the turquoise and brown modules were positively associated with 24h urinary protein, aPLA2Rab levels, PMN stages, IgG deposition, and glomerular sclerosis (*P*<0.05, **Figure 3G**). Within subcluster 0 plasma cells, genes within the brown module displayed intriguing correlations. They positively correlated with 24hour urinary protein levels and PMN stages, while inversely correlating with IgG deposition and glomerular sclerosis (**Figure 3G**). In all plasma cells subsets, genes within the blue module were negatively correlated with 24h urinary protein, serum aPLA2Rab levels and PMN stages (*P*<0.05, **Figure 3G**). Of interest, we found that genes within the turquoise and brown modules were greatly up-expressed in subcluster 0 (**Figure 3H**). Furthermore, the UMAP visualizations demonstrated that the turquoise and brown modules were predominantly localized within subcluster 0, underscoring their unique affiliation with this specific subgroup (**Figure 3I**). Genes within turquoise, blue, and brown modules were majorly enriched in ribosome, cell cycle process, and protein targeting to membrane-related pathways, respectively (**Figure 3J**). The identified modules demonstrate interconnectivity, forming intricate regulatory networks through various interaction mechanisms (**Figure 3J**). Taken together, it may suggest that the occurrence of subcluster 0 plasma cells, as well as increased expression of genes in turquoise or brown modules may aggravate the occurrence and progression of PMN.

### The role of regulatory B cells in the pathogenesis and progression of PMN

**Figure 4:**
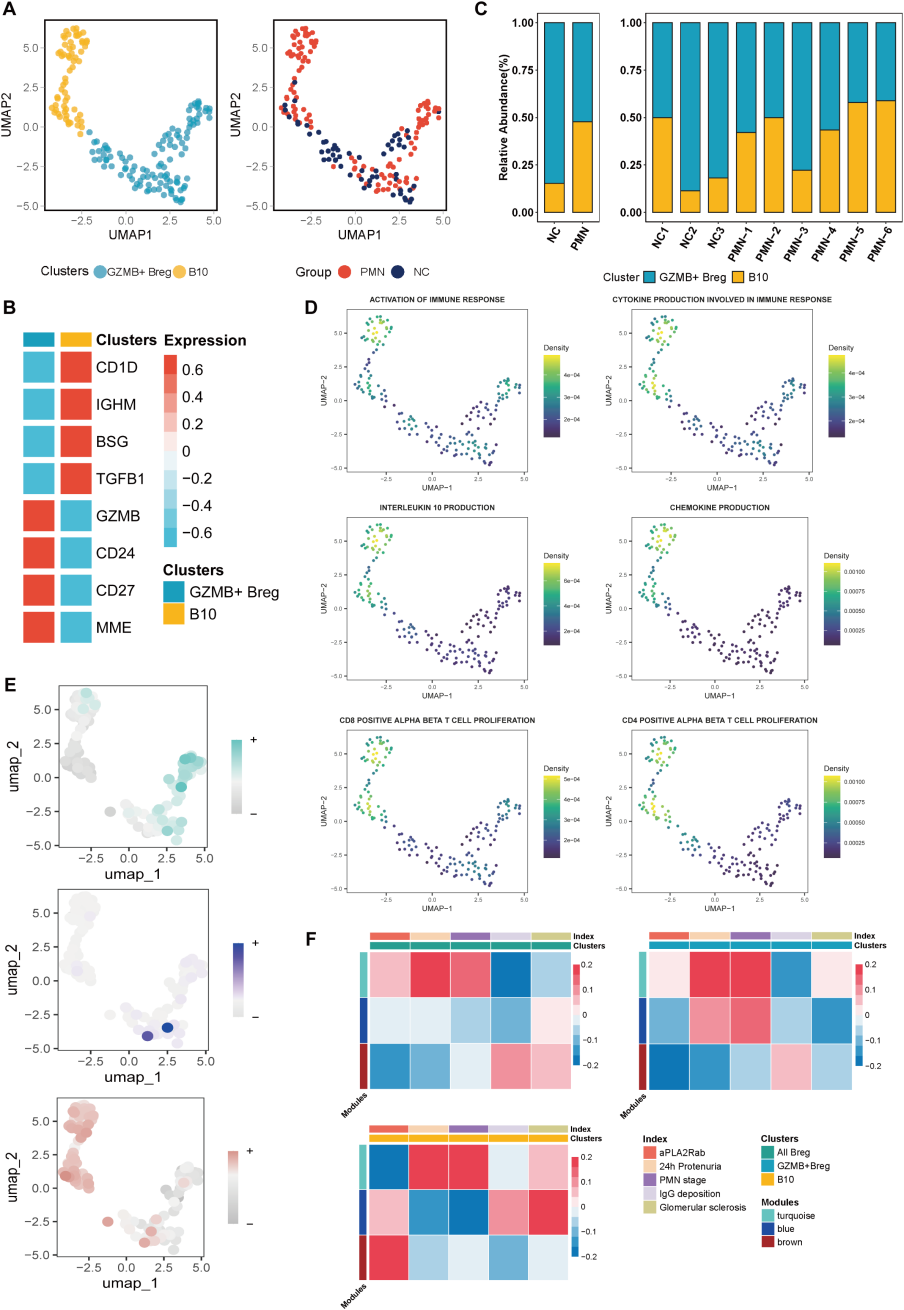
Characterization of Breg cells subclusters. **(A)** Unsupervised clustering analysis for Breg cells. **(B)** Heatmap of selected marker genes in different subsets of Breg cells. **(C)** The stacked bar chart showing the abundance of different Breg cells subclusters between groups and individuals. **(D)** UMAP plot of cell pathway score in Breg cells. **(E)** UMAP plot of module scores in Breg cells of modules identified previously in plasma Cells. **(F)** Heatmap of correlation between the three modules and clinical index in Breg cells. aPLA2Rab, anti-phospholipase A2 receptor antibody; hME, harmonized module eigengenes.

In our investigation into the role of Breg cells in PMN pathogenesis and development, we uncovered two distinct subpopulations. GZMB+ Breg cells were identified as being marked by the expression of GZMB, CD24, CD27, MME, while B10 cells showed elevated levels of CD1D, IGHM, BSG, and TGFβ1 (**Figures 4A, B**). Compared with NC, the PMN group had a higher proportion of B10 cells (15.2% vs 47.8%) (**Figure 4C**). B10 cells demonstrated significantly stronger functions in terms of immune response activation, CD4 and CD8 αβ T cell proliferation, chemokine production, cytokine production involved in immune responses, and interleukin-10 production, compared to GZMB^+^ Breg cells (**Figure 4D**, P *<*0.05). Next, we evaluated the expression of genes within the three modules identified previously and the correlation with clinical parameters in Breg cells. Genes in the turquoise module were mainly expressed in GZMB^+^ Breg cells, which were positively associated with 24h urinary protein and PMN stages (*P*<0.05, **Figures 4E, F**). As mainly expressed in B10 cells, genes within the brown module were positively associated with serum aPLA2Rab levels (*P*<0.05, **Figures 4E, F**). Results of hdWGCNA in Breg cells further confirmed the role of genes within turquoise and brown modules in the pathology and development process of PMN.

### Dynamic trajectories of B cells throughout PMN progression

**Figure 5:**
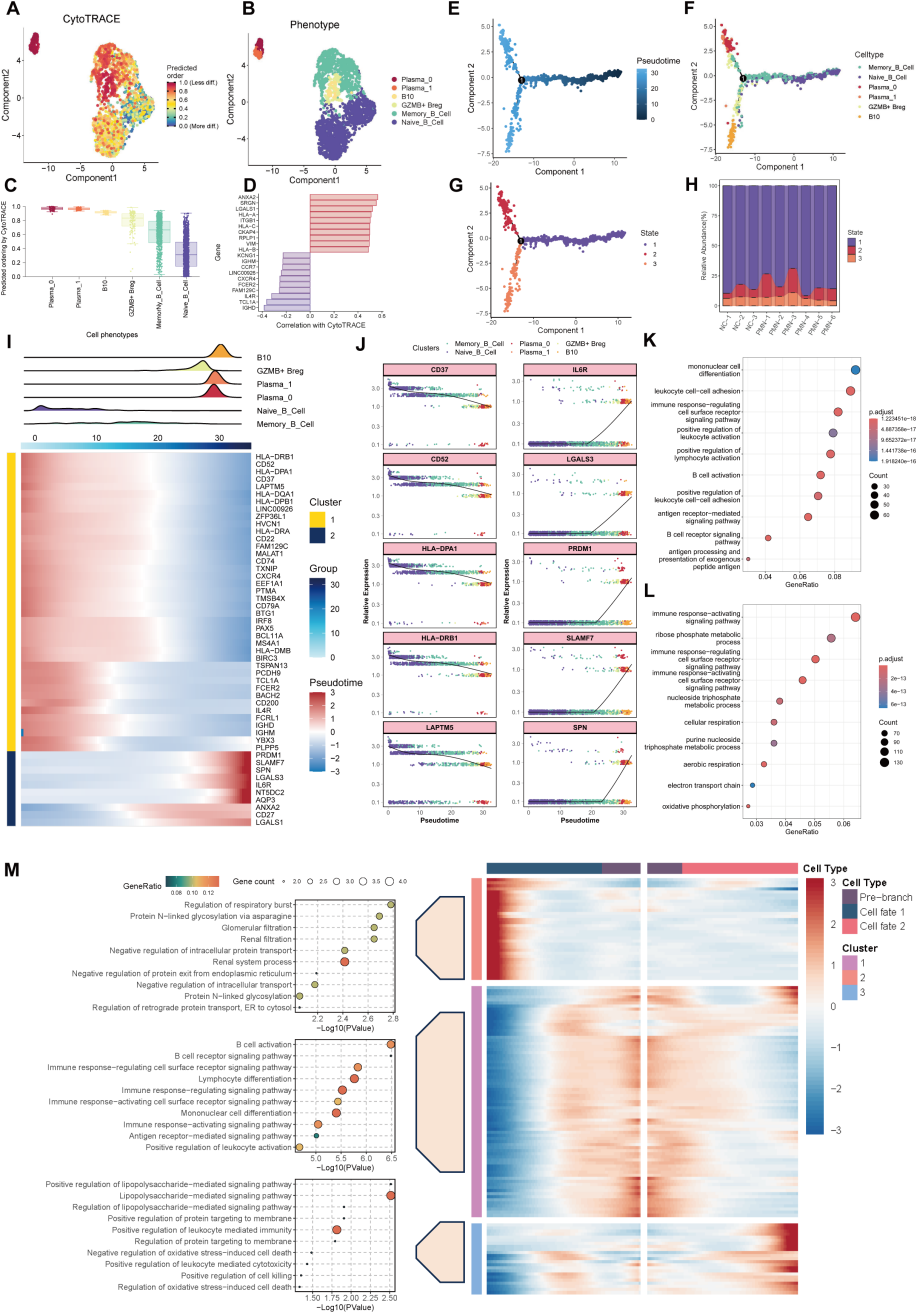
Trajectory analysis of B cells. **(A)** UMAP plot depicting the distribution of CytoTRACE scores among B cells. Dark-red indicates lower scores (low stemness) while dark-blue indicates higher scores (high stemness). **(B)** UMAP plot labels the B cells by different phenotype. **(C)** Inferred stemness order of B cell subsets using CytoTRACE. **(D)** The bar chart showed the genes related to the cells with the highest degree of differentiation and the lowest degree of differentiation according to the correlation with CytoTRACE. **(E, F)** Developmental trajectories of B cells inferred by monocle2 using DDR-Tree and default parameter, each cell subtype marked with a different color. **(G)** Different states of B cells differentiation. **(H)** The stacked bar chart showing the abundance of different states of B cells between groups and individuals. **(I)** Cell density variation of B cell subtypes during the pseudotime (top), pseudo-heatmap of the representative genes with different expression patterns. **(J)** Pseudo-scatter plots showing the expression variation and distribution of some specific genes during the pseudotime, color-coded by cell types. **(K, L)** GO enrichment analysis of genes re-clustered into 2 clusters. **(M)** pseudo-heatmap of representative genes in differentiation branches by BEAM analysis (right). Cell fate 1 represent the branch to plasma cells and Cell fate 1 represent branch to Breg cells. GO functional enrichment analysis of genes re-clustered into 3 clusters (left). GO, Gene Ontology; BEAM, branch expression analysis model.

CytoTRACE and monocle2 methodology were employed to prognosticate and assign scores to cellular stemness in diverse cells, thereby providing insights into the temporal order of cell state transitions within the context of PMN. The data demonstrate that plasma cells and Breg cells emerged as the ultimate differentiation trajectory for naïve B cells (**Figures 5A–H**). Based on distinct expression patterns across the trajectory, genes were clustered into two clusters (**Figure 5I**). Cluster 1 were expressed specifically in the early developmental stages, including CD37, CD52, HLA-DPA1, HLA-DRB1, LAPTM5, etc. They were enriched in B cell activation, B cell receptor signaling, and antigen procession and presentation pathways (**Figures 5J, K**). Besides, as expressed in the late developmental stages, genes in cluster 2 such as IL6R, LGALS3, PRDM1, SLAMF7, and SPN, were mainly associated with immune response activation-related pathways (**Figures 5J, L**), indicating the activation of B cells and immune response involved in the progression of PMN. Furthermore, this trajectory was unfolded sequentially with three distinct states: the initial pre-branch point, as well as two subsequent branches known as Cell fate 1(plasma cells) and Cell fate 2 (Breg cells) by BEAM analysis. Beam genes were identified and clustered into 3 expression patterns. Cluster 1, cluster 2, and cluster 3, were expressed in pre-branch cells, plasma cells, and Breg cells, respectively. Further GO enrichment analysis of genes associated with pseudotime revealed distinct functional profiles for these clusters. Genes in Cluster 2 were primarily involved in “regulation of glomerular filtration” processes, while genes in Cluster 1 focused on “activation of B cell”. Genes in Cluster 3 exhibited enrichment for functions related to “regulation of protein targeting to membrane” (**Figure 5M**).

### Altered cell-cell communications of PBMC in PMN patients

**Figure 6:**
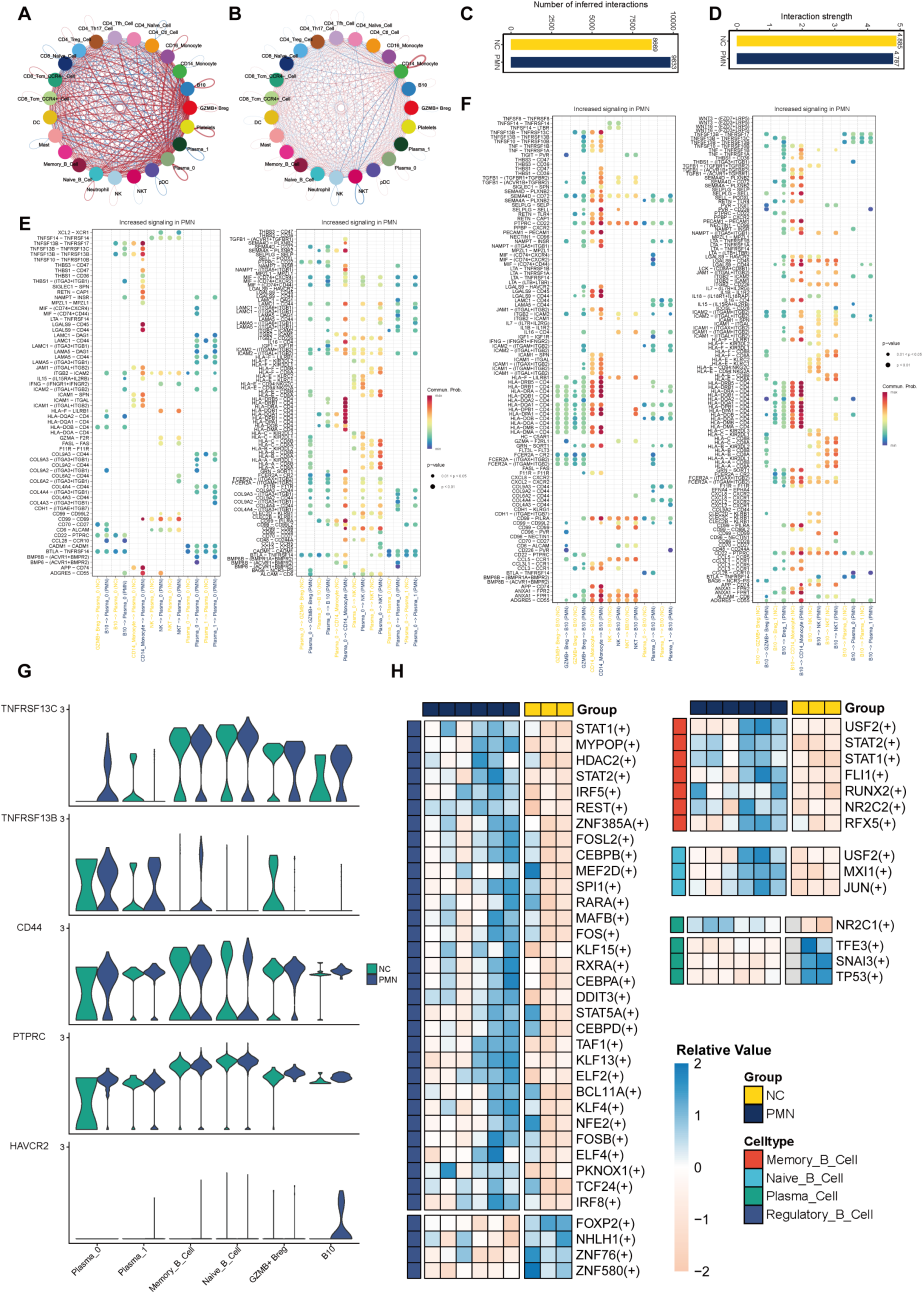
Altered cell–cell interactions in PBMC cells and differential expressed transcription factors. **(A-D)** Comparisons of overall changes in cell-cell communication between PMN patients and normal controls, including the differential number of interactions **(A, C)** and differential interaction strength **(B, D)** between immune cell. **(E, F)** Communication probabilities of up-regulated ligand-receptor pairs between subcluster 0 of plasma cells, subcluster 1 of Breg cells, and other immune cells, with the dot color reflecting the communication probability, blank indicating the communication probability zero, and dot size representing the p value. **(G)** Violin plot of genes TNFRSF13C, TNFRSF13B, CD44, PTPRC, and HAVCR2 expression level in B cells. **(H)** Heatmap of differential activity transcription factors in B cell subsets. SCENIC analysis was performed using utilizing pySCENIC. Limma was used to identify differential TFs activity between MN and NC groups with adjusted p value < 0.05 and absolute log2fold-change > 0.5. SCENIC, Single-cell regulatory network inference and clustering.

To decipher the dynamics of cellular responses, intercellular crosstalk and the altered signaling pathways in patients with PMN, we analyzed the scRNA-seq datasets of PBMC using CellChat cell communication analysis. The results revealed that PBMC in PMN established extensive communication networks with various cell types, setting them apart from those in NC (**Figures 6A, B**). The increased complexity (number of interactions) and decreased intensity (strength of interactions) of intercellular networks may underscore the transformative nature of PMN (**Figures 6C, D**). Then the up-regulated ligand-receptor pairs between subcluster 0 of plasma cells, B10 cells, and other immune cells under the PMN environment were further explored. B cell activating factor (BAFF), galectin, and major histocompatibility complex class II (MHC-II) information flows were markedly increased in patients with PMN (**Figures 6E, F**). Specifically, TNFSF13B (i.e. BAFF) and LGALS9 (i.e. galectin-9) were identified as secreted by CD14^+^ monocytes communicating with subcluster 0 of plasma cells and B10 cells. In response, plasma cells and B10 cells through MHC-II information flow such as HLA-DRB5-CD4 ligand-receptor pair to interact with CD14^+^ monocyte (**Figures 6E, F**). Expression analysis reinforced these cell communications. TNFSF10, TNFSF13B, and LGALS9 were mainly expressed in CD14^+^ monocyte (**Supplementary Figure S2B**), with the increased expression of BAFF receptors like TNFRSF13C (i.e. BAFF receptor), TNFRSF13B (i.e. TACI), TNFRSF13C (i.e. BCMA), TNFRSF10B, and LGALS9 receptors like PTPRC, HAVCR2 in subcluster 0 of plasma cells and B10 cells (**Figure 6G**, **Supplementary Figures S3A, B**).

### Transcriptional factors of B cells in PMN

By using SCENIC analysis, the top 3 TFs with major transcriptional regulatory roles in naïve B cells were FOXP, ZNF43, ZNF131. In memory B cells, TCF7L1, HIVEP1, and MEF2C were the major regulatory TFs. The most relevant specific regulators of plasma cells were FOXD4, FOXD1, and HNF1B. In Breg cells, BCL11B, ZNF385A, and MAFB were identified as important regulatory TFs (**Supplementary Figures S3C, D**). By differential expression analysis, we identified 47 differentially active TFs in PMN patients (**Figure 6H**, **Supplementary Table S2**). Most TFs with altered activity were identified in Breg cells (*n* = 35). Functional analysis revealed these TFs participated in B cell activation, B cell differentiation, regulation of inflammatory response, and regulation of cytokine production pathways (**Supplementary Table S3**). STAT1 and STAT2 were found to be activated in both Breg cells and memory B cells, whereas USF2 was activated in naïve B cells and memory B cells. NR2C1 was uniquely upregulated TF in plasma cells of PMN, and its potential functional role was shown to be associated with cell pluripotency maintenance. These TFs were displayed as promising candidates for promoting inflammatory responses and immune reaction, which may contribute to the progression of PMN.

### Association of serum galectin-9 levels with disease activity of PMN

**Figure 7:**
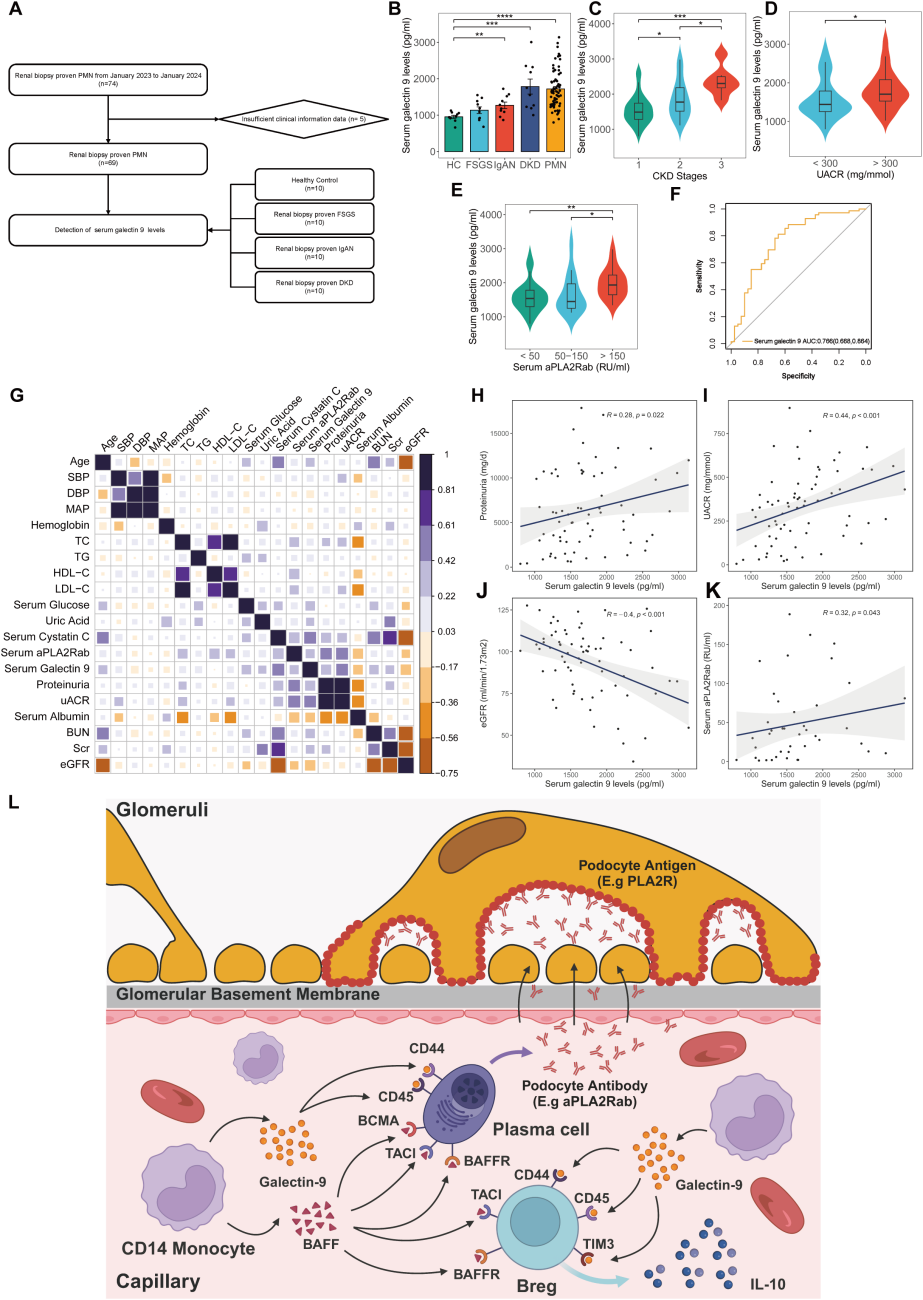
Clinical significance of serum galectin-9 level in kidney diseases. **(A)** Flowchart of study participants. **(B)** Comparison of serum galectin-9 levels in healthy control, patients with FSGS, IgAN, DKD, and PMN. **(C)** Comparison of serum galectin-9 levels in PMN patients with different CKD stages. **(D)** Comparison of serum galectin-9 levels in PMN patients with different UACR. **(E)** Comparison of serum galectin-9 levels in PMN patients with different serum aPLA2Rab. Comparisons between groups were made using one-way analysis of variance (ANOVA), Kruskal-Walli’s test, or χ2 test as appropriate. **(F)** ROC curves for the diagnostic performance of serum galectin-9 for discriminating PMN from non-PMN. **(G-K)** Spearman correlation analysis between serum galectin-9 levels and clinical parameters in PMN patients. **(L)** Schematic illustration of the mechanism of galetin-9 in PMN. HC, healthy control; FSGS, Focal segmental glomerulosclerosis; IgAN, IgA nephropathy; DKD, Diabetic kidney disease; PMN, Primary membranous nephropathy; aPLA2Rab, anti-phospholipase A2 receptor antibody; UACR, urine albumin-to-creatinine ratio; eGFR, Estimated glomerular filtration rate.

The BAFF pathway has been implicated in the pathogenesis of PMN, contributing to disease progression by fostering immune complex formation and inducing podocyte injury (21). Monoclonal antibodies targeting this pathway have demonstrated efficacy in autoimmune diseases such as systemic lupus erythematosus, prompting an investigation into the potential therapeutic application in PMN (22). However, the significance of the galectin pathway, particularly galectin-9, within the context of PMN remains largely uncertain. We investigated the role of serum galectin-9 level in the diagnosis and pathogenesis of PMN. A total of 69 patients diagnosed with PMN, 10 patients with FSGS, 10 patients with IgAN, 10 patients with DKD, and 10 HC were enrolled (**Figure 7A**). Patients with PMN had significantly higher levels of serum galectin-9 compared to HC, FSGS, and IgAN groups (PMN vs HC: *P*< 0.001; PMN vs FSGS: *P*< 0.001; PMN vs IgAN: *P* = 0.006). Besides, serum galectin-9 levels were also significantly higher in the IgAN and DKD groups when compared with HC group (*P*=0.036, *P*< 0.01, respectively **Figure 7B**). In PMN group, there was a significant decrease in serum galectin-9 levels at stage G1 relative to later CKD stages (G1 vs G3: *P*< 0.001; G1 vs G2: *P* = 0.046; G2 vs G3: *P* = 0.036, **Figure 7C**). Moreover, serum galectin-9 levels were significantly elevated in patients with higher UACR (UACR>300 vs. <300 mg/mmol, *P*=0.027, **Figure 7D**). Furthermore, galectin-9 levels rise across the increasing levels of aPLA2Rab (<50 RU/ml vs. >150 RU/ml: *P*< 0.001; 50–150 RU/ml vs. >150 RU/ml: *P* = 0.041, **Figure 7E**). While elevated levels of galectin-9 were also found in patients with IgAN and DKD, serum galectin-9 demonstrated a higher AUC of 0.766 for distinguishing PMN patients from non-PMN individuals (sensitivity = 81.2%, specificity = 65%, **Figure 7F**). On further analyses by Spearman test, the serum galectin-9 levels were positively correlated with 24h urinary protein (r = 0.346, *P*=0.022, **Figures 7G, H**), UACR (r = 0.443, P < 0.001, **Figures 7G, I**), and serum aPLA2Rab (r = 0.324, *P* = 0.043, **Figures 7G, K**), whereas it was negatively correlated with eGFR (r = -0.367, *P* < 0.001, **Figure 7J**). Compared with the low serum galectin-9 group (<1643 pg/ml), patients with a high level of serum galectin-9 (>1643 pg/ml) had higher levels of 24h proteinuria, UACR, serum creatinine, serum BUN, FBG, serum Cystatin C (*P <*0.05), but lower levels of albumin and eGFR (*P <*0.05, **Table 2**).

**Table 2:** Baseline clinicopathological characteristics stratified by different serum galectin 9 levels.

Parameter	Total (n=69)	Low serum galectin 9 group (<1643 pg/ml, n=34)	High serum galectin 9 group (>1643 pg/ml, n=35)	P value
Age (years)	53.29 ± 13.02	50.79 ± 13.39	55.71 ± 12.36	0.118
Gender (male/female)	47/22	23/11	24/11	1
Clinical parameter				
SBP (mmHg)	134.12 ± 16.1	132.65 ± 14.37	135.54 ± 17.72	0.458
DBP (mmHg)	85.35 ± 9.67	87.56 ± 9.39	83.2 ± 9.58	0.061
Laboratory parameter				
Urinary protein (mg/d)	5890 (2901, 10493)	3877(1540, 6552)	6592 (4175, 10791)	**0.007**
UACR (mg/mmol)	330 ± 203	248 ± 194	410 ± 182	**< 0.001**
eGFR (ml/min/1.73 m²)	100 (80, 108)	104 (91, 112)	90 (73, 102)	**0.006**
BUN (mmol/L)	5.18 (4.08, 6.83)	4.71 (4.07, 5.31)	5.75 (4.56, 7.88)	**0.037**
Scr (μmol/L)	71 (57, 81)	70 (54, 75)	73 (63, 90)	**0.036**
Uric acid (mmol/L)	373 ± 85	358 ± 84	387 ± 84	0.162
Serum albumin (g/L)	24.6 (21.3, 28.3)	26.75 (23, 31.45)	23.2 (19.75, 25.2)	**0.004**
FBG (mmol/L)	4.29 ± 0.71	4.07 ± 0.63	4.5 ± 0.73	**0.011**
TG (mmol/L)	2.15 (1.61, 2.67)	1.98 (1.58, 2.65)	2.4 (1.7, 2.68)	0.365
TC (mmol/L)	6.87 (6.06, 8.95)	6.64 (5.8, 8.69)	7.06 (6.12, 9.15)	0.319
LDL-C (mmol/L)	4.41 (3.68, 5.50)	4.07 (3.67, 5.67)	4.61 (3.70, 4.98)	0.359
HDL-C (mmol/L)	1.43 (1.20, 1.83)	1.45 (1.21, 1.82)	1.42 (1.17, 1.79)	0.732
Hemoglobin (g/L)	135 ± 18	139 ± 19	137 ± 17	0.566
Serum Cystatin C (mg/L)	1.02 (0.86, 1.25)	0.92 (0.82, 1.02)	1.18 (1, 1.42)	**< 0.001**
aPLA2Rab (RU/ml)	35.2 (2.1, 121.8)	19.5 (1.9, 56.0)	42.6 (10.6, 221.1)	0.094
Serum galectin 9 (pg/ml)	1644(1340, 1952)	1330 (1217, 1463)	1953(1761, 2352)	**< 0.001**
Medications				
CNI (%)	25 (36)	14 (41)	11 (31)	0.554
CD20 (%)	19 (28)	8 (24)	11 (31)	0.642
CTX (%)	1(1)	0 (0)	1 (3)	1
Expectant treatment (%)	15 (22)	11 (32)	4 (11)	**0.007**
CNI & CD20 (%)	9 (13)	1 (3)	8 (23)	**0.028**

SBP, systolic blood pressure; DBP, diastolic blood pressure; eGFR, estimated glomerular filtration rate; BUN, blood urea nitrogen; Scr, serum creatine; FBG, fasting blood glucose; TG, triglyceride; TC, total cholesterol; LDL-C, low-density lipoprotein cholesterol; HDL-C, high-density lipoprotein cholesterol; aPLA2Rab, serum anti-phospholipase A2 receptor antibody.

Data were presented as the mean ± standard, the median with interquartile range or counts and percentages. A two-tailed p<0.05 was considered statistically significant. Bold value: p<0.05.

## Discussion

The current study depicts an immune system atlas that analyzes the immunocyte compositions, cell–cell communication, gene expression, and the dominating pathways from 42,653 immune cells in PBMC of patients with PMN and NC at a single-cell resolution. A total of 11 major immune cell subsets were revealed. Neutrophils and plasma cells exhibited significantly different between PMN and NC. Moreover, our results provided evidence for the changes in the critical B cell subsets in PMN, such as subsets 0 plasma cells and B10 Breg cells. Cell-cell communication analysis suggests that the BAFF pathway and galectin-9 pathway might be potential molecules that regulate the activity of plasma cells and Breg cells (**Figure 7L**). Correspondingly, we validated that serum galectin-9 levels were significantly higher in PMN group compared to other glomerulonephritis groups or HC. Besides, the levels were significantly associated with kidney function.

Neutrophils, infiltrating immune cell commonly observed in the kidneys of glomerulonephritis patients, contribute to microvascular damage by promoting apoptosis and prolonging their presence within the glomeruli (23). Neutrophil activation, indicative of chronic low-grade inflammation, contributed to the propagation of venous thromboembolism (24). Besides, Neutrophil extracellular traps could initiate and foster immunothrombosis (24). Our results revealed significantly higher proportions of neutrophils in patients with PMN compared to NC. Combined with neutrophils’ role in promoting coagulation, partially explains the hypercoagulable state observed in PMN.

It is widely recognized B cells are the key drivers of PMN. The study by Gu et al. found that B cells were increased in PBMC of PMN group vs. NC which was in disagreement with our study (11). The baseline serum aPLA2Rab levels of the patients may explain the difference as the median aPLA2Rab level was relatively lower in our study compared to that in Gu’s study (11). Moreover, limited samples between both studies may also contribute to the discrepancy (six PMN patients in our study vs. three PMN patients in Gu’s study). These observations call for further large PMN cohorts to validate and to allow a novel insight of B cells in the clinical setting. In our study, while individual B cell subsets displayed unique gene expression patterns, substantial overlap in their DEGs was also observed. RPS26, a ribosomal subunit structural protein involved in growth and development, was increased in memory B cells, naïve B cells, and plasma cells of PMN patients. This finding aligns with previous research reporting upregulation of RPS26 in the mesangial cells of IgAN compared to healthy donors, as observed in a single-cell survey (25). Further investigation into the function of RPS26 in PMN pathogenesis is warranted.

Plasma cells represent the terminal stage in B cell differentiation, specialized for antibody production (26). In patients with PMN, plasma cells produce autoantibodies (e.g. aPLA2Rab) that bind to the antigens on the surface of podocyte (e.g. PLA2R), forming immune complexes deposited on the GBM. These complexes activate the complement system, triggering inflammation and further damaging renal tubules and podocytes (1). Consistent with previous studies, the proportion of plasma cells was found a significant increase in PMN compared to NC (4, 11). Plasma cells also exhibited the most significant differential gene expression compared with NC, as revealed by a pseudobulk method designed to minimize false positives. RPS6KA5, a substrate of the MAPK-activated protein kinase family, was upregulated in plasma cells of patients with PMN. The upregulation of RPS6KA5 may be involved in inducing humoral immune responses and contributing to the production of autoantibodies (27). Conversely, TMMP9 expression was decreased in PMN patient plasma cells. TMMP9 degrades collagen IV, a major component of basement membranes, and plays a crucial role in glomerular pathology (28). Additionally, BUD31, HIST1H4C, and ATPAF1 were significantly upregulated in patients with PMN. Although these genes have been linked to metabolism, the roles in PMN pathogenesis remain unclear (29–31). The enrichment of ATP hydrolysis-related genes among DEGs of plasma cells suggests a functional shift in these cells during PMN pathogenesis. Furthermore, our findings of increased protein secretion in plasma cells from PMN patients strengthen the understanding of circulating autoantibodies in pathogenesis and highlight the potential for plasma cell-targeted therapies in PMN. Further unsupervised clustering analysis revealed a distinct plasma subcluster characterized by high expression of the marker gene RPS10. We designated this subcluster as “cluster 0”. Cluster 0 plasma cells were almost exclusively derived from patients with PMN. Cluster 0 cells displayed a significantly high level of positive autoimmune antibodies and exhibited heightened autoimmunity potential. Beyond their autoimmune activities, cluster 0 plasma cells also modulate neutrophil function, highlighting their broader immunomodulatory capacity. Cluster 0 cells also showed increased expression of ITPR3, a receptor and calcium channel responsible for facilitating calcium release from the endoplasmic reticulum (ER) (32). ITPR and its isoforms directly mediate the transfer of Ca^2+^ from the ER via ITPR, influencing ATP production and cell survival, which are crucial for mitochondrial Ca^2+^ signaling (33). To elucidate the connections between gene expression modules in plasma cells and clinical characteristics, we employed hdWGCNA analysis. Our results revealed a significant correlation between genes within brown and turquoise modules and clinical characteristics such as 24h proteinuria, serum aPLARab levels. While these modules mainly focus on ribosomes and transport proteins to the cell membrane process. The activation of these process reflects broader metabolic shifts within plasma cells during PMN, specifically an increase in autoantibody production illustrated above. Currently, there are limited studies on subcluster 0 plasma cells in the context of kidney diseases or autoimmune conditions, highlighting the need for further research into their unique role and function within autoimmune process. Targeting cluster 0 plasma cells or related pathways may represent a promising avenue for future pathogenesis-based treatments in patients with PMN.

Breg cells, widely recognized for their immunomodulatory functions and suppressive effects on the immune system, can differentiate from various stages of B cell development (34). In our study, we observed a higher number of differentially expressed TFs and DEGs in Breg cells compared to other cell types, suggesting a potentially critical role for these cells in PMN pathology. Chiara et al. found that MN patients display significantly higher percentages of circulating Breg than patients with non–immune-mediated CKD and healthy controls (35). Similar findings are also reported in IgAN patients (36). In the contrast, the results from Raja et al. showed that PMN patients had a lower percentage of Breg cells compared to healthy controls, and those who responded to therapy saw a recovery in their Breg population (37). Thus, dynamic fluctuations in Breg cell populations constitute a regulatory mechanism controlling alloreactive immunity, adapting to the overall immune status. DEGs of Breg cells also revealed intriguing changes. GREM1, a developmental gene reactivated in response to kidney injury, was identified among these DEGs. Recent studies have highlighted its role in renal fibrosis progression, suggesting it as a potential therapeutic target for diabetic kidney disease and crescentic immune-mediated glomerulonephritis (38–40). MYOM2 expression, conversely, was decreased in Breg cells from PMN patients. The deficiency could lead to significant downregulation of CD2AP and synaptopodin (41). Furthermore, HLA-DQA2, previously reported as a highly expressed gene in PMN and involved in the most enriched pathway, exhibited cell-specific expression patterns (25), aligning with our findings. Elevated IFN-γ signaling in both plasma and Breg cells suggests a self-amplifying cycle. T cells secrete pro-inflammatory IFN-γ, stimulating plasma and Breg cells, and further drive the innate immune cascade, contributing to oxidative stress and inflammation in PMN (42, 43).

Breg cells can be divided into different subgroups based on their phenotypic and functional characteristics. In our study, Breg cells were divided into two major subsets, GZMB^+^ Breg cells and B10 cells. B10 cells have been reported to inhibit pathological autoimmune response by secreting interleukin-10, transforming growth factor-β (TGF-β), and adenosine and through other ways to prevent T cells and other immune cells from expanding (34, 44). In HBV-associated membranous nephropathy (HBV-MN), the percentage of B10 cell and IL-10 levels were significantly higher than in healthy controls. These levels negatively correlated with 24h proteinuria and positively correlated with renal function (45). However, in IgAN patients, B10 cells and its intracellular expression of IL-10 were lower than healthy controls. And the percentage of B10 cells was negatively correlated with the level of serum galactose deficient-IgA1 (Gd-IgA1) (36). GZMB+ Breg cells, which could be induced by IL-21, act its function mainly through granzyme B (GrB). GZMB+ Breg cells can efficiently suppress T-cell proliferation by GrB-dependent TCR-ζ degradation. GZMB+ Breg cells also may contribute to the modulation of cellular adaptive immune responses by Treg-like mechanisms (46). The significant reduction of GZMB+ Breg cells observed in autoimmune diseases such as SLE, contrasted with their increase in transplant patients with renal graft tolerance, underscores the pivotal role these cells play in maintaining immune homeostasis (47, 48). Our results revealed that B10 cells were increased in patients with PMN, and displayed showed stronger immunoregulatory effects than GZMB+ Breg cells. Thus, more attention may be better paid on B10 cells of Breg cells, pointing to a new direction for the discovery of the pathogenesis of PMN.

Cell-cell communication analysis suggests that the BAFF pathway and galectin-9 pathway may play a role in regulating the activity of subcluster 0 plasma cells and B10 cells, potentially through interactions with CD14 monocytes. Our findings corroborate Gu et al.’s observation of enhanced plasma cell BAFF signaling within PBMC (11). BAFF, a key survival factor for B cells, belongs to the tumor necrosis factor ligand family (49). It’s expressed by various cell types, including monocytes, dendritic cells, and bone marrow stromal cells (49, 50). BAFF exerts its effects through three distinct receptors: TNFRSF13B/TACI (transmembrane activator and calmodulin cyclin ligand interaction factor), TNFRSF17/BCMA (B-cell maturation antigen), and TNFRSF13C/BAFFR. These receptors are predominantly found on immune cells of the B cell lineage (51). Elevated BAFF levels in autoimmune patients favor plasma cell persistence, resulting in a continuous surge of autoantibodies that directly contribute to kidney damage (52–54). Previous research has consistently demonstrated significantly elevated circulating BAFF levels in IgA nephropathy, systemic lupus erythematosus, and PMN patients compared to healthy controls (52–54). Notably, these higher BAFF levels correlate positively with the severity of renal injury (52–54). One clinical study found that serum levels of BAFF and APRIL correlated with serum aPLA2Rab levels and predicted clinical response in aPLA2Rab-positive PMN (54). Furthermore, therapeutic strategies focused on BAFF have yielded remarkable success in treating these kidney diseases. Belimumab, a recombinant human monoclonal antibody, directly targets and inhibits BAFF. This targeted approach has proven effective in significantly reducing proteinuria in patients with systemic lupus erythematosus and improving their renal prognosis (55). Telitacicept, a TACI fusion protein that targets both BAFF and a proliferation-inducing ligand (APRIL), has emerged as an effective treatment for IgA nephropathy. It specifically reduces circulating galactose-deficient-IgA1 levels and alleviates proteinuria in patients (56, 57). In addition to its effectiveness in managing IgA nephropathy, recent studies indicate that Telitacicept can also reduce proteinuria in HIV infection-related aPLA2Rab-positive PMN (58). Therefore, these findings provide new insights into the potential of BAFF signaling as a reliable disease-activity biomarker and treatment target in PMN, which will be an interesting subject of future studies.

Galectin-9 is a protein with diverse roles, including participation in cell-cell communication, signaling, adhesion, migration, proliferation, and apoptosis (59). Galectin-9 plays a significant role in shaping immune responses. It acts as a chemoattractant for eosinophils, influences neutrophil chemotaxis, and enhances phagocytosis (60). In monocytes, galectin-9 triggers the production of proinflammatory cytokines IL-1α, IL-1β, and IFN-γ, but it also promotes the development of immunosuppressive macrophages (61). Additionally, studies have demonstrated that galectin-9 induces apoptosis in Th1 and Th17 cells while stimulating Treg activity (62). Galectin-9 exerts a potent inhibitory effect on B cell activation and function. It suppresses B cell receptor (BCR)-mediated activation (63, 64) and human cord blood-derived stem cells directly regulate activated B cells through a galectin-9 mediated mechanism, leading to decreased proliferation and altered phenotypes (65).The significance of galectin-9 in kidney diseases is becoming increasingly clear. Galectin-9 administration attenuates kidney disease severity in murine lupus models by suppressing toll-like receptor 7–mediated activation of plasmacytoid dendritic cells and B cells (66). In type 2 diabetes, increased serum galectin-9 correlates with declining GFR (67), while recombinant galectin-9 therapy in mouse models mitigates glomerular hypertrophy, albumin excretion, and TGF-β expression (68). Research also reveals that galectin-9 plays a crucial role in protecting against acute kidney injury. In LPS-induced and ischemia-reperfusion (IR) induced acute kidney injury models, galectin-9 levels surged in plasma and kidney (69, 70). Notably, mice lacking galectin-9 suffered significantly worse kidney damage (70). Combining delayed ischemic preconditioning with galectin-9 therapy proved effective in mitigating IR injury by reducing proinflammatory cell infiltration and boosting the presence of anti-inflammatory cells (71). Furthermore, galectin-9 exhibits a protective effect in anti-glomerular basement membrane glomerulonephritis (anti-GBM GN). Administration of galectin-9 in anti-GBM GN mice delays the rise in serum creatinine, reduces renal tubular injury, and minimizes crescent formation. This protection is linked to decreased Th1 and Th17 cell infiltration in the kidney due to suppressed expression of inflammatory chemokines. Concurrently, galectin-9 promotes a Th2 immune response, contributing to its overall beneficial effect in anti-GBM GN (72). Our study revealed significantly increased serum galectin-9 levels in PMN vs. IgAN, FSGS and HCs. And the increased levels directly correlated with kidney function, suggesting a crucial role for galectin-9 in PMN pathogenesis. Our findings pave the way for exploring galectin-9 as a therapeutic target to enhance treatment efficacy, the underlying mechanism of which needs to be further explored.

Our study acknowledges several limitations that warrant consideration. Firstly, the relatively small sample size may not fully capture the diversity of disease severity and stages in PMN. A larger cohort is needed to ensure better representativeness. Secondly, our findings of PMN are currently based on transcriptomic analysis alone. Further investigations utilizing BCR/TCR sequencing are necessary to comprehensively assess changes in cell surface molecules. Thirdly, the absence of a post-treatment group due to the challenges associated with sample collection limits our understanding of B cell responses to PMN therapies. Fourthly, although our findings demonstrate increased galectin-9 levels in PMN, several important questions remain unanswered. Future investigations are required to elucidate the specific regulatory mechanisms by which galectin-9 influences B cells in this context, and whether its levels change in response to treatment or disease remission. Finally, validation of these novel findings through tissue staining, *in vitro* functional studies using cell lines or primary human cells, and animal models of PMN could offer more useful clues for strengthening the robustness of our conclusions.

Taken together, this study reveals novel insights into the heterogeneity and functional diversity of critical B cell subsets within PMN at single-cell resolution. Specifically, we identified distinct roles for subset 0 plasma cells and B10 Breg cells in PMN pathogenesis. Furthermore, our findings implicate galectin-9 as potential regulator and treatment target of this autoimmune disease. These discoveries pave the way for developing targeted therapies that modulate B cell function and hold promise for improved treatment outcomes in PMN.

## Data Availability

Raw data, processed data, and metadata from human single-cell data-sets have been deposited in the repository NCBI GEO with the accession number GSE283826. The source code used to analyze the scRNA-seq data in the current study is available online at the GitHub repository (https://github.com/bigfang-lab/scRNA-PBMC-PMN.git).

## Supplementary material

Supplementary Figure 1 Distribution of samples and clusters of PBMC, and alterations of B cells in patients with PMN. **(A)** UMAP plot for 42653 PBMC cells, unsupervised clustered with 19 clusters. **(B)** UMAP plot for 42653 PBMC cells, colored by sample source. **(C)** Upset plot of DEGs of each B cells subsets. **(D)** UMAP plot of the expression of RPS26 and AC010422.3 between PMN and normal control groups. **(E, F)** The top 6 activated pathways in PMN memory B cells and naïve B cells identified via GSVA analysis. **(G-J)** UMAP plot of cell pathway score in B cells between PMN and normal control groups.

Supplementary Figure 2 Pathways score in plasma cells. **(A-D)** UMAP plot of cell pathway score in plasma cells.

Supplementary Figure 3 BAFF, galectin-9 expressions, and TF activity of B cells. **(A)** Violin plot of genes TNFSF10, TNFSF13B, and LGALS9 expression levels in PBMC cells. **(B)** Violin plot of genes TNFRSF17 and TNFRSF10B expression levels in B cells subsets between PMN and normal control groups. **(C, D)** Specificity scores of regulons in B cells subsets. The top 3 TFs with higher activity are noted.

Supplementary Figure 4 Distribution of T cell subset. **(A)** UMAP plot of distribution of the T cells subsets. **(B)** Heatmap of selected marker genes in different subsets of T cells.

Supplementary Table 1 Differentially expressed genes in B cells.

Supplementary Table 2 Differentially active TFs between PMN and NC in B cells.

Supplementary Table 3 GO enrichment analysis of TFs.
