# Supplementary figures and images for: Exploring immunological alterations of B cells in peripheral immunity via single-cell RNA sequencing: insights into primary membranous nephropathy
Source: Front Immunol. 2025 Aug 19;16:1622395. doi: 10.3389/fimmu.2025.1622395 (PMC12401916; doi:10.3389/fimmu.2025.1622395)

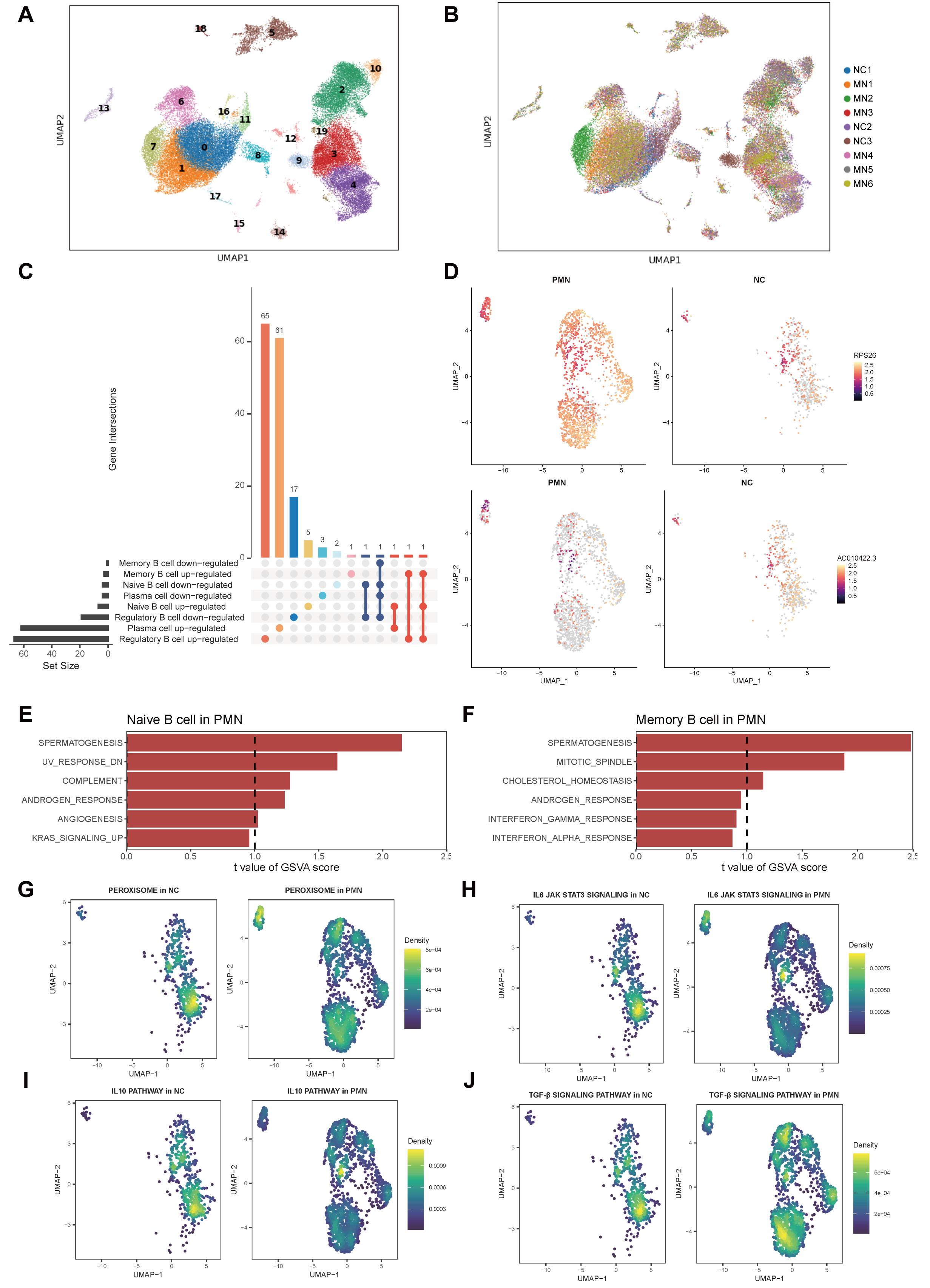

Supplement: Supplementary Figure 1 — Distribution of samples and clusters of PBMC, and alterations of B cells in patients with PMN. (A) UMAP plot for 42653 PBMC cells, unsupervised clustered with 19 clusters. (B) UMAP plot for 42653 PBMC cells, colored by sample source. (C) Upset plot of DEGs of each B cells subsets. (D) UMAP plot of the expression of RPS26 and AC010422.3 between PMN and normal control groups. (E, F) The top 6 activated pathways in PMN memory B cells and naïve B cells identified via GSVA analysis. (G-J) UMAP plot of cell pathway score in B cells between PMN and normal control groups. [file Image1.tif]

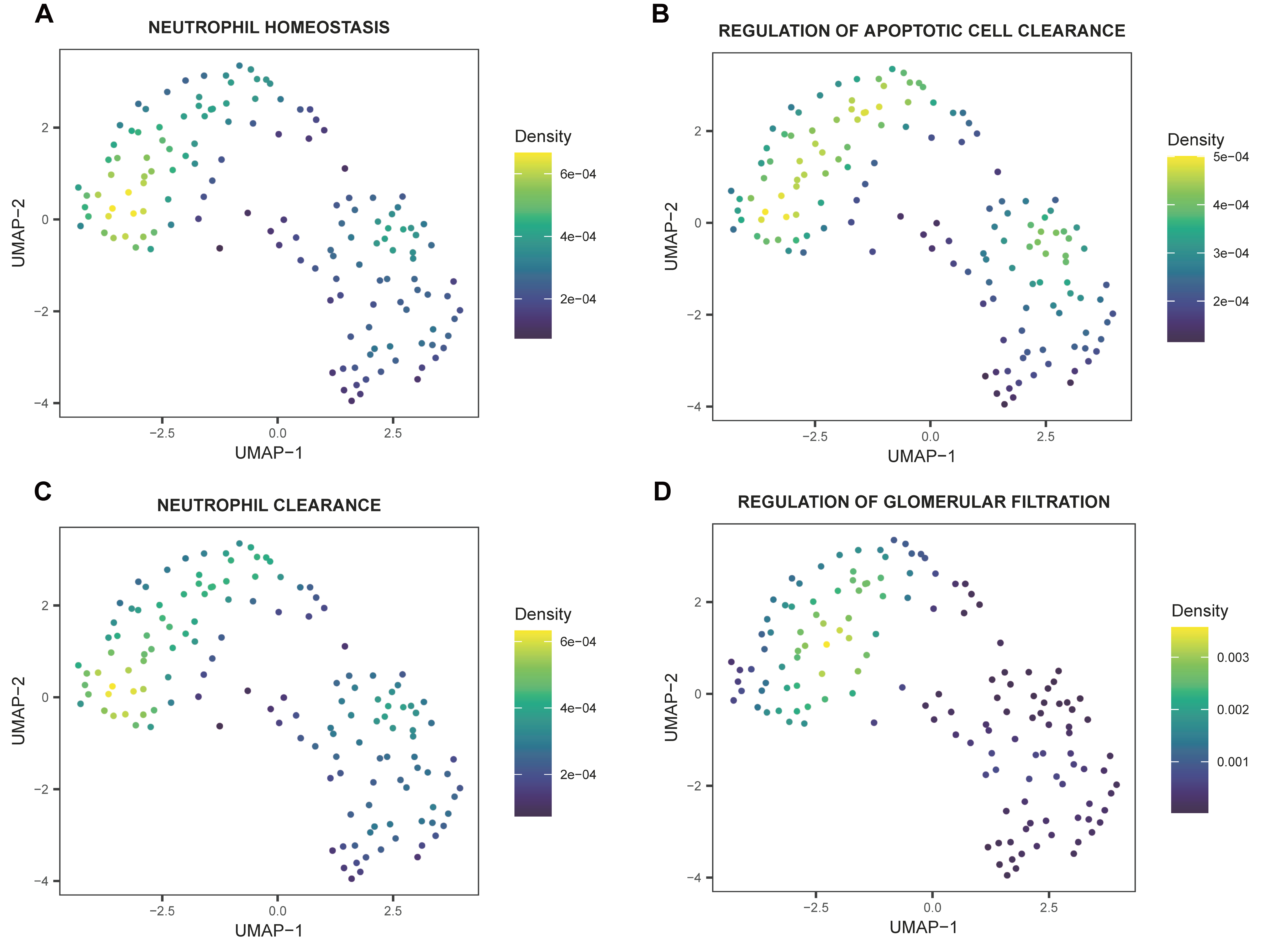

Supplement: Supplementary Figure 2 — Pathways score in plasma cells. (A-D) UMAP plot of cell pathway score in plasma cells. [file Image2.tif]

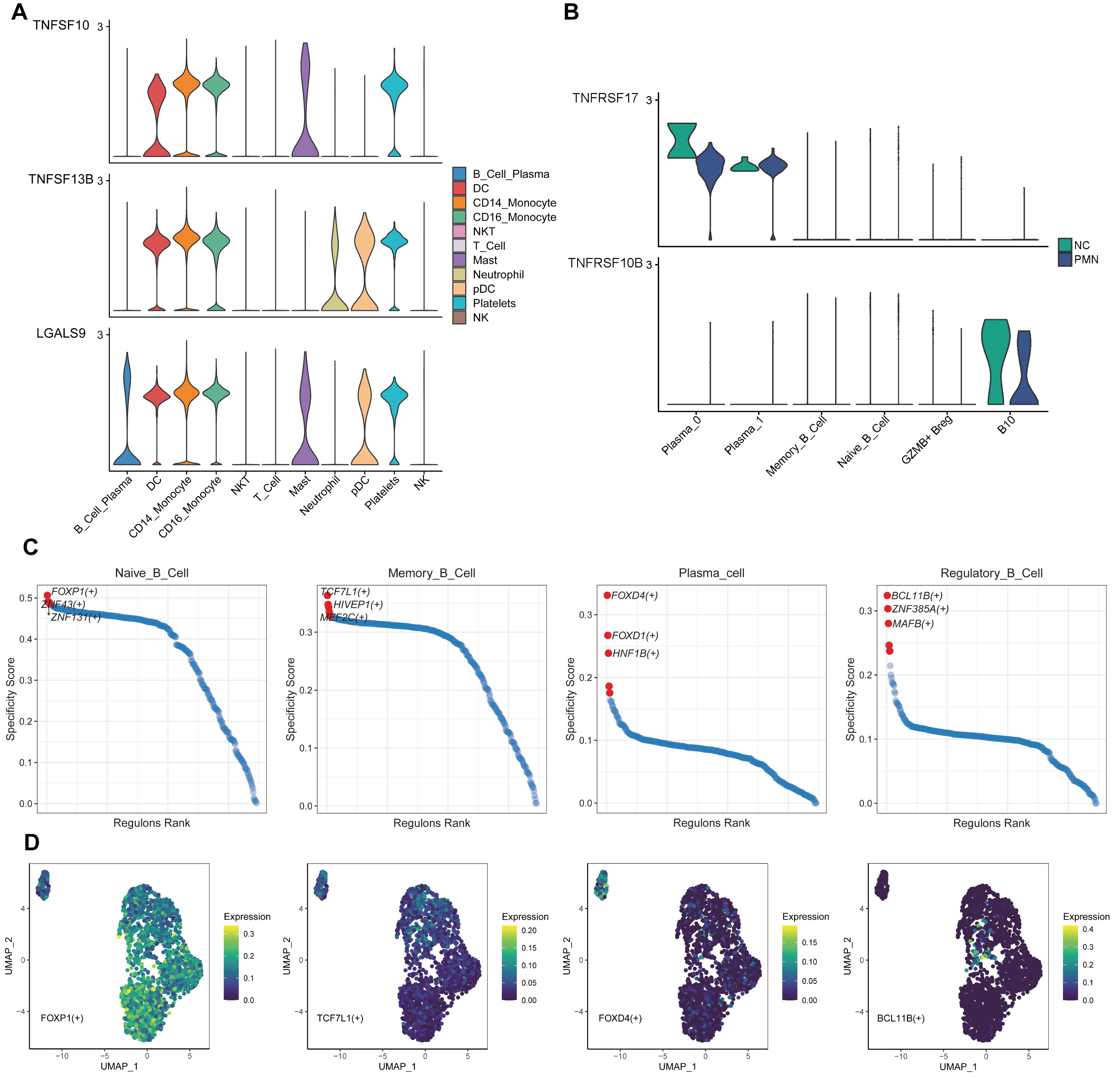

Supplement: Supplementary Figure 3 — BAFF, galectin-9 expressions, and TF activity of B cells. (A) Violin plot of genes TNFSF10, TNFSF13B, and LGALS9 expression levels in PBMC cells. (B) Violin plot of genes TNFRSF17 and TNFRSF10B expression levels in B cells subsets between PMN and normal control groups. (C, D) Specificity scores of regulons in B cells subsets. The top 3 TFs with higher activity are noted. [file Image3.tif]

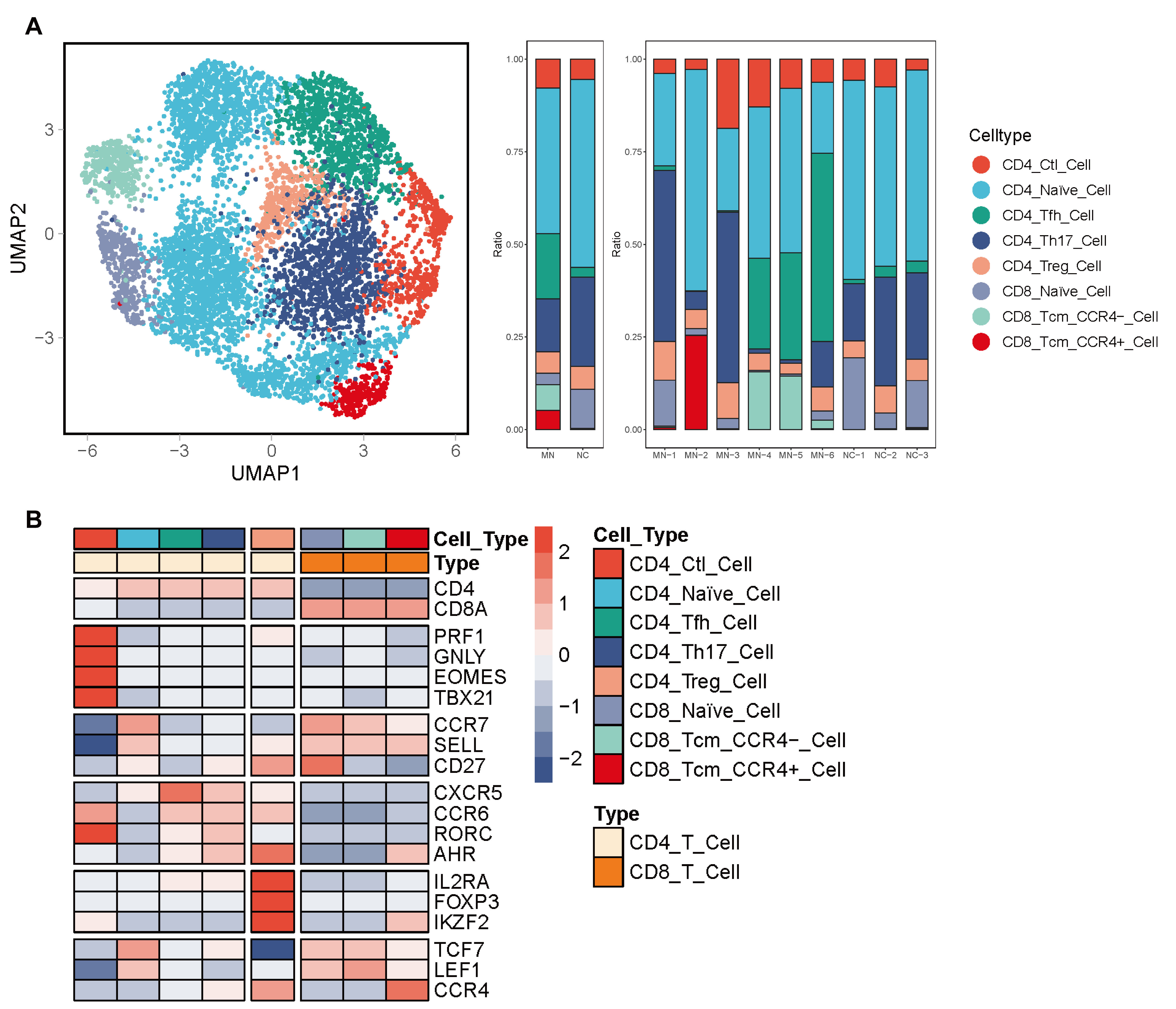

Supplement: Supplementary Figure 4 — Distribution of T cell subset. (A) UMAP plot of distribution of the T cells subsets. (B) Heatmap of selected marker genes in different subsets of T cells. [file Image4.tif]
